# Executive function in adolescents with PKU and their siblings: Associations with biochemistry

**DOI:** 10.1016/j.ymgmr.2015.08.001

**Published:** 2015-08-24

**Authors:** R. Sharman, K. Sullivan, R. Young, J. McGill

**Affiliations:** a University of the Sunshine Coast, ML 32 Maroochydore DC, QLD 4558, Australia; b Queensland University of Technology, Kelvin Grove, Brisbane, QLD 4059, Australia; c Department of Metabolic Medicine, Lady Cilento Children's Hospital, Brisbane, QLD, Australia

**Keywords:** EF, executive function, Phenylketonuria, Adolescents, Phenylalanine:tyrosine ratio, Executive function, Dopamine

## Abstract

Previous research shows consistent and marked executive function impairment in children with early and continuously treated phenylketonuria. This between groups analysis (phenylketonuria group vs sibling controls) found no significant differences in executive function (although adolescents with phenylketonuria performed slightly worse than their siblings). Biochemical relationships with executive function were confined to long-term measures of high phenylalanine:tyrosine ratio exposure, as well as tyrosine exposure independent of phenylalanine. This study suggests that early and continuously treated PKU results in non-significant EF differences (compared to siblings), although the influence of long-term exposure to poorer metabolic control is still evident.

## 1 Introduction

The functional impact of executive function (EF) impairments in children with early and continuously treated phenylketonuria (ECT-PKU) has been well documented and quite marked, leading to an increased risk of ADHD diagnosis [1], [2], [7]. This study replicates previous investigations exploring the relationship between EF impairment and biochemical control in this population [6]. Consistent with previous research it was expected that 1) children with PKU would show greater impairments than their siblings in EF, and that EF impairment would be more strongly associated with poorer metabolic control (higher phenylalanine, phenylalanine:tyrosine ratio and lower tyrosine markers; [3]).

## 2 Method

### 2.1 Participants

Thirteen children with classical PKU were recruited as part of a larger study from Royal Children's Hospital, Brisbane, Australia. All children have ECT-PKU and Table 1 presents their biochemical data. The characteristics of the 13 children with classical PKU were: seven male and six female and mean age of 13.95 years (SD: 1.8 years; range: 10.26 years to 16.26 years). Nine siblings (6 male; 3 female) were assessed for between groups analyses. Their mean age at testing was 13.12 years (SD: 3.4 years; range: 7.5 years to 17.58 years). Five siblings were older than their sibling with PKU, and six were of the same gender.

### 2.2 Materials

The Behaviour Rating Inventory of Executive Function (BRIEF) provides an ecologically valid measure of the manifestation of EF deficits in daily life e.g., impact on school work, family functioning and social relationships [4]. The BRIEF was recommended by the Waisbren and White [8] review as an appropriate test instrument for this population.

The Global Executive Composite (GEC; an overall score of EF impairment) as well as working memory subscale were used in the analyses. Working memory (WM) was also chosen given the consistent findings that it remains an executive function most at risk in this cohort [2].

### 2.3 Procedure

Parents filled out the BRIEF questionnaire concurrent with neuropsychological test administration as part of a larger study whilst on-site at the Royal Children's Hospital.

### 2.4 Scoring

Raw scores were converted to t-scores to account for variations in age and sex. A t-score of 50 represents average function, with a deviation of 10 representing the equivalent of one standard deviation; higher t-scores indicate higher levels of impairment. A deviation of 15 (i.e., a t-score of 65 or above) is considered by the BRIEF scoring manual as clinically significant and in need of further investigation.

## 3 Results

An independent samples t-test was conducted to test for significant differences in GEC and WM between children with classical PKU and their siblings and no significant difference was found (Table 2).

### 3.1 Biochemical markers associated with BRIEF t-scores

Correlations between lifetime, < 12 year, and concurrent measures of phenylalanine, tyrosine, and the phe:tyr ratio as well as the two measures of EF were generated to test for associations between biochemical markers in the classical PKU cohort and parent report of EF impairment (GEC and WM). t-Scores were used to account for age and sex variations. One-tailed Pearson's *r* was used to assess significance in all correlations given the directional nature of the hypotheses. Three significant correlations were observed: lifetime tyrosine and GEC (*r* = −.546, *p* = .027), tyrosine < 12 years and GEC (*r* = −.500, *p* = .041) and lifetime phenylalanine:tyrosine ratio and GEC (*r* = .478, *p* = .049). These correlations indicated that lower lifetime tyrosine and lower tyrosine prior to 12 years were associated with an increased parent report of global EF impairment, and that a higher lifetime phenylalanine:tyrosine ratio was associated with an increased parent report of global EF impairment. WM showed no significant associations with biochemistry; likewise no measure of phenylalanine showed any significant associations with EF.

## 4 Discussion

In this sample, the BRIEF did not detect significant differences between children with classical PKU and their siblings, although t-scores indicate descriptively higher (worse) EF in the PKU group compared to their siblings. Given the small sample size our study was likely underpowered, previous PKU-sibling research using larger samples has shown small but statistically significant differences (in the region of one third of a standard deviation worse), and our data demonstrates similar differences [5], [9].

Three significant correlations between children with PKU and their phenylalanine:tyrosine ratio and tyrosine independent of phenylalanine were observed. The failure to a) find significant between-groups differences and b) significant correlations on the WM scale is likely due to the small sample size. Correlations between tyrosine and EF impairment were in the expected direction (negative), in that lower levels of lifetime (and < 12 years) tyrosine were associated with higher levels of EF impairment. Although not all correlations between tyrosine and GEC/WM were statistically significant, all tyrosine correlations were in a negative direction, further indicating a trend towards low levels of tyrosine associated with increased EF impairment.

A high lifetime phenylalanine:tyrosine ratio was also found to correlate positively with GEC, in that the higher the ratio, the higher the level of parent-reported impairment. No correlations between phenylalanine on its own and EF were observed in this sample. This study has also found an association between tyrosine levels on their own (i.e., independent of phenylalanine) and EF.

In all, these results provide support that poorer metabolic control underpins EF impairment in this population [3]. Phenylalanine exposure was not shown to be associated with EF deficit in this sample, rather, the strongest associations between biochemistry and reported EF, involved long-term tyrosine deficit or a high phenylalanine:tyrosine ratio. This is likely because the sample was early and continuously treated, with reasonable phenylalanine compliance across their lifetime. Nonetheless, subtle influences of poorer metabolic control on EF remained evident.

## Acknowledgements and conflicts of interest

The authors would like to thank the Royal Children's Hospital Foundation for their generous grant (10242; Allied Health Research Project Grant) worth $24,491. The authors have no conflicts of interest to report.

## Figures and Tables

**Table 1:** Means (SDs) and ranges of biochemical markers of 13 children with classical PKU.

	Phenylalanine mean (SD) and range	Tyrosine mean (SD) and range	Phe:tyr ratio mean (SD) and range
Lifetime	438 (149)	97 (17)	7.4 (4.2)
226–735	67–121	3.6–19.5
< 12 years	415 (146)	95 (15)	7.1 (4.1)
226–706	67–114	3.6–18.9
Concurrent	713 (273)	105 (40)	7.9 (4.4)
200–1200	45–170	1.3–16

**Table 2:** Differences in BRIEF T scores between children with classical PKU and sibling controls.

	Mean	SD	Significance
PKU — WM	60.1	14.3	
Sibling — WM	56.0	12.2	*p* = .511
PKU — GEC	56.1	11.8	
Sibling — GEC	53.1	8.1	*p* = .542

Note: WM — working memory and GEC — global executive composite.
